# Genome-wide expression analysis of reactive oxygen species gene network in Mizuna plants grown in long-term spaceflight

**DOI:** 10.1186/1471-2229-14-4

**Published:** 2014-01-06

**Authors:** Manabu Sugimoto, Youko Oono, Oleg Gusev, Takashi Matsumoto, Takayuki Yazawa, Margarita A Levinskikh, Vladimir N Sychev, Gail E Bingham, Raymond Wheeler, Mary Hummerick

**Affiliations:** 1 Institute of Plant Science and Resources, Okayama University, 2-20-1 Chuo, Kurashiki, Okayama 710-0046, Japan; 2 National Institute of Agrobiological Sciences, 2-1-2 Kannondai, Tsukuba, Ibaraki 305-8602, Japan; 3 Institute of Fundamental Medicine and Biology, Kazan Federal University, Kazan 420008, Russia; 4 Institute of Space and Astronautical Science, JAXA, Tsukuba, Ibaraki 305-8505, Japan; 5 Agriculture, Forestry and Fisheries Research Council, Ministry of Agriculture, Forestry and Fisheries, 1-2-1 Kasumigaseki, Chiyoda-ku, Tokyo 100-8950, Japan; 6 Hitachi Government & Public Corporation System Engineering Ltd, 2-4-18 Toyo, Koto-ku, Tokyo 135-8633, Japan; 7 Institute of Biomedical Problems, Russian Academy of Sciences, 76a Khorosheevskoe shosse, Moscow 123007, Russia; 8 Space Dynamics Laboratory, Utah State University, 1695 North Research Park Way, Logan, Utah 84341-1942, USA; 9 Kennedy Space Center, NASA, Florida 32899, USA

**Keywords:** mRNA-Seq, Next generation sequencing, Transcriptome, Mizuna, Reactive oxygen species, International Space Station, Spaceflight

## Abstract

**Background:**

Spaceflight environment have been shown to generate reactive oxygen species (ROS) and induce oxidative stress in plants, but little is known about the gene expression of the ROS gene network in plants grown in long-term spaceflight. The molecular response and adaptation to the spaceflight environment of Mizuna plants harvested after 27 days of cultivation onboard the International Space Station (ISS) were measured using genome-wide mRNA expression analysis (mRNA-Seq).

**Results:**

Total reads of transcripts from the Mizuna grown in the ISS as well as on the ground by mRNA-Seq showed 8,258 and 14,170 transcripts up-regulated and down-regulated, respectively, in the space-grown Mizuna when compared with those from the ground-grown Mizuna. A total of 20 in 32 ROS oxidative marker genes were up-regulated, including high expression of four hallmarks, and preferentially expressed genes associated with ROS-scavenging including thioredoxin, glutaredoxin, and alternative oxidase genes. In the transcription factors of the ROS gene network, MEKK1-MKK4-MPK3, OXI1-MKK4-MPK3, and OXI1-MPK3 of MAP cascades, induction of WRKY22 by MEKK1-MKK4-MPK3 cascade, induction of WRKY25 and repression of Zat7 by Zat12 were suggested. RbohD and RbohF genes were up-regulated preferentially in NADPH oxidase genes, which produce ROS.

**Conclusions:**

This large-scale transcriptome analysis revealed that the spaceflight environment induced oxidative stress and the ROS gene network activation in the space-grown Mizuna. Among transcripts altered in expression by space conditions, some were common genes response to abiotic and biotic stress. Furthermore, certain genes were exclusively up-regulated in Mizuna grown on the ISS. Surprisingly, Mizuna grew in space normally, as well as on the ground, demonstrating that plants can acclimate to long-term exposure in the spaceflight environment by reprogramming the expression of the ROS gene network.

## Background

Plant cultivation in space will be necessary to augment stored foods when space mission distances and durations increase, such as for long term bases on the Moon and Mars, and even being considered for orbiting bases such as the International Space Station (ISS). Plants can play an important role in supplying nutrients, oxygen, and water to humans and can be co-utilized for waste recycling in space [1,2], therefore, establishment of the culture approaches and breeding/developing plants to optimize their performance in space will be critical to future advanced space life support systems. In space, plants can be exposed to unusual and extreme environments, such as space radiation, reduced gravity, reduced atmospheric pressure, elevated or super-elevated CO_2_ concentrations, and temperature excursions. In orbital, spaceflight cabin, lunar and Martian bases, environmental conditions for plant cultivation such as temperature, light, air, and water could be controlled, however, it would be hard to eliminate space radiation and reduced gravity, which are suspected to perturb plant growth and development.

Space radiation and microgravity are suggested to generate reactive oxygen species (ROS) in plants. Ionizing radiation produced ROS [3] and increased lipid peroxidation products in erythrocytes of mice housed for about 100 days in ISS [4], and exposure of *Arabidopsis* seedlings and callus cells to altered gravitational forces (clinorotation or hyper gravity) up-regulated genes associated with cellular signaling, protein phosphorylation/dephosphorylation, defense, stress response, and gravisensing [5-7]. Gene expression profiles have been analyzed to understand the stress level and adaptation challenges in plants grown in space. *Arabidopsis* plants (7 days old) launched and grown for 5 days in the Space Shuttle showed that the genes of HSP, drought-inducible calcium binding protein, a MADS-box protein, and MAP kinases were induced 4-fold, compared with those of ground control [8]. Pathogen response, wounding, drought, and cold associated genes, a gene associated with auxin-mediated lateral root development, and an essential regulator gene of photorespiration were up-regulated more than five-fold in the *Arabidopsis* seedlings, which were germinated and grown for 12 days in the Space Shuttle. In addition, genes associated heat shock, salt, drought, metals, wounding, phosphate, ethylene, senescence, terpenoids, seed development, cell walls, photosynthesis, and auxin were up-regulated by more than five-fold in the culture cells grown for 12 days in the Space Shuttle [9]. These results indicate that plant genes in the ROS network, which are induced by abiotic and biotic stress [10,11], can be triggered by changes related to the spaceflight environment. However, because of the limitation of launching and cultivation in space, few studies on gene expression profiles have been performed, and in many cases plant materials were transported to orbit (i.e., not grown entirely in spaceflight), subjecting them to transient responses that may not have been due to spaceflight environment.

We have developed a plant growth system, namely Lada, which was installed in ISS to study and grow plants, including vegetables in a spaceflight environment. We have succeeded in cultivating Mizuna, tomato, pea, radish, wheat, rice, and barley in Lada aboard ISS. Both M1 and M2 pea grown from seeds formed during spaceflight showed no significant difference in growth, developmental characteristics, frequency of chromosome aberrations in primary root meristem, and level of molecular polymorphism between offspring of space-grown and ground control seeds [12]. On the other hand, transcription levels of superoxide dismutase, glutamyl transferase, catalase, and ascorbate peroxidase were increased in the barley germinated and grown for 26 days in Lada, though the whole-plant growth and development of the barley in spaceflight were the same as in the ground control barley [13]. These results suggest that plants can adapt to spaceflight environment by changing their ROS gene network.

In this study, we investigated the response of the ROS gene network in Mizuna, *Brassica rapa* var. *nipposinica*, cultivated for 27 days in ISS. Mizuna is one of the model plants for cultivating in Lada aboard ISS because it is a leafy vegetable that can fully develop in 4 weeks in order to supply not only a plant material for investigating the effect of spaceflight environment but also a fresh vegetable for astronauts to take nutrient and benefit mental health quickly [14]. The ROS gene network consists of a vast numbers genes, therefore, large-scale transcriptome analysis is necessary to clarify the stress level and adaptation challenges in plants under the spaceflight environment. The mRNA-Seq strategy using next generation sequencing (NGS) technology has become a powerful tool for analyzing genome-wide gene expression and global transcriptional networks. mRNA-Seq overcomes the limitations of microarray analysis, of which the probes are designed on the basis of a reference genome sequence of model species and cover only small portion of a gene. We demonstrated that an overall gene expression of the ROS gene network in the space-grown Mizuna was characterized by mapping reads from the NGS analysis to the *Brassica* database as a reference genome sequence and annotating genes. Our results provide a survey of plant response to the spaceflight environment and define common and different genes and cascades in the ROS gene network for adapting to the spaceflight environment.

## Results and discussion

### Growth of Mizuna in space

The seeds of Mizuna was transported to ISS, germinated, and grown in “Lada”, plant growth chamber aboard the Russian segment of ISS, which has successfully produced a harvest of fresh vegetables and seeds in space [12,14,15]. The fresh weight and water content of the space-grown Mizuna were 82.9 g and 92.5%, and those of the ground-grown Mizuna were 58.0 g and 92.0%, respectively, after 28 days of cultivation, showing that Mizuna grew as well or better under spaceflight environment in ISS as well as on the ground. The reduced growth of the ground controls was likely due to the close matching of watering protocols for space and ground plants, which resulted in slower germination on the ground and some wilting symptoms of some ground control plants just prior to harvest. Although similar amounts of water were being added to space and ground plants, the tendency of water to settle to the bottom of the root trays on the ground (and be more uniformly distributed in space) could have affected overall growth.

### Construction of the Mizuna transcript dataset and identification of differentially expressed transcripts

Total reads of transcripts from the space-grown and the ground-grown Mizuna by paired-end sequencing assay were 73,028,136 and 74,578,954. Also, 44,994,328 (61.6%) and 49,195,408 (66.0%) of transcripts of total reads were mapped to the reference *Brassica rapa* genome sequence, respectively. A G-test [false discovery rate (FDR) < 0.001] was performed on the read count of transcripts to detect differences in gene expression between the space-grown and the ground-grown Mizuna, and to identify responsive transcripts of the space-grown Mizuna. The numbers of up-regulated and down-regulated transcripts were 8,258 and 14,170, which respectively included 467 and 1,208 unannotated transcripts (Additional file 1: Table S1).

**Figure 1:**
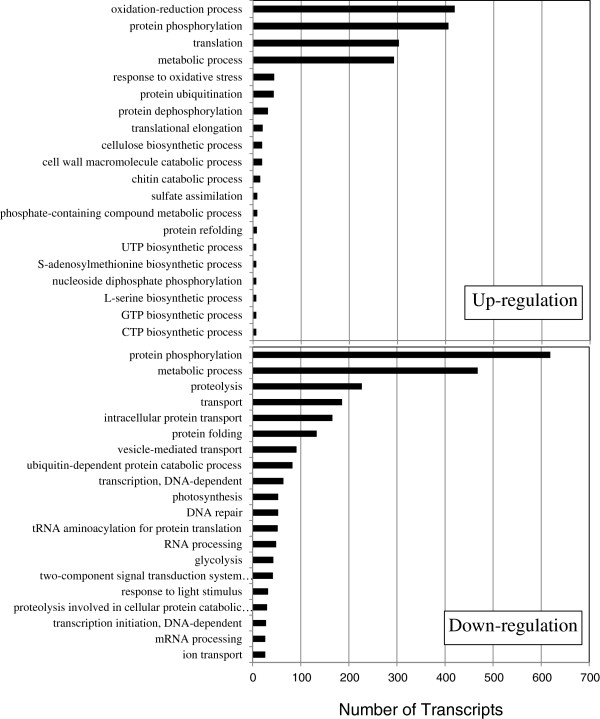
**Distribution of up-regulated and down-regulated genes in biological process category of Gene Ontology.** A total of 1,707 and 2,895 probe sets of up-regulated and down-regulated genes respectively in the space grown Mizuna were assigned to the biological category. The number of top 20 GO terms in up-regulated genes and down-regulated genes are shown. The *x*-axis and *y*-axis indicate the number of transcripts and the category, respectively.

These responsive genes were functionally characterized using Gene Ontology (GO) enrichment analysis. A total of 1,707 and 2,895 probe sets of up-regulated and down-regulated genes were respectively assigned to 26 and 52 terms in biological process category. The greatest increase in up-regulated genes occurred in oxidation-reduction process (GO:0055114), followed by protein phosphorylation (GO:0006468), the total of which represented 48% of biological process, whereas that of down-regulated genes was protein phosphorylation (GO:0006468), which represents 38% (Figure 1, Additional file 2: Table S2). Cytochrome P450 and protein kinase were the main genes in oxidation-reduction process and protein phosphorylation, respectively. Cytochrome P450 is associated with the detoxification reactions of xenobiotic compounds activated to electrophilic molecules to maintain the redox homeostasis [16]. Protein kinases play key roles in signal transduction under abiotic and biotic stress. These results suggest that redox-oxidation state was perturbed and that signal transduction is regulated in the cells of the space-grown Mizuna.

### Expression profiles of oxidative marker and ROS-scavenging genes

Table 1 shows 32 ROS oxidative marker genes up-regulated more than five-fold by hydrogen peroxide, superoxide, or singlet oxygen in *Arabidopsis*, including putative trypsin inhibitor (At2g43510), unknown protein (At1g19020), unknown protein (At1g05340), transmembrane receptor (At1g57630), and unknown protein (At2g21640), which are regarded as hallmarks for the general oxidative stress response [17]. In the space-grown Mizuna, 20 of 32 genes were up-regulated more than five-fold. Especially, 4 of 5 hallmark genes, Bra04768 (At2g43510, putative trypsin inhibitor), Bra031061 and Bra025707 (At1g19020, unknown protein), Bra015419 (At1g05340, unknown protein), Bra031207 (At2g21640, unknown protein), showed a greater than ten-fold increase in expression. These results show that the spaceflight environment induces oxidative stress in the cells of space-grown Mizuna.

**Table 1:** Expression of oxidative marker genes in the space-grown Mizuna

**Gene name**	**AGI code**	***B. rapa *** **gene**	**Fold change**
Trypsin inhibitor, putative	At2g43510	Bra004768	34
		Bra037705	1.8
		Bra014534	0.01
Disease resistance protein putative	At1g57630	ND	
Expressed protein	At1g19020	Bra031061	41
		Bra025707	19
Expressed protein	At1g05340	Bra015419	43
Expressed protein	At2g21640	Bra031207	13
Embryo-abundant protein-related	At2g41380	Bra016937	12
DNAJ heat shock	At3g08970	Bra029736	2.2
		Bra036657	0.47
Glutathione S-transferase, putative	At2g29490	ND	
WRKY family transcription factor	At1g62300	Bra027057	5.8
		Bra034482	1.1
Glutathione S-transferase, putative	At2g29470	Bra039983	6.1
tolB protein-related	At4g01870	Bra000913	1.8
Embryo-abundant protein-related	At4g22530	Bra013623	2.1
Glutathione S-transferase, putative	At1g17170	Bra025995	11
Armadillo/beta-catenin repeat family protein	At3g09350	Bra029761	7.3
		Bra001314	1.4
UDP-glucosyl transferase family protein	At2g43820	Bra000330	11
		Bra000329	1.1
		Bra004787	0.68
UDP-glucosyl transferase family protein	At3g11340	ND	
Cell division cycle protein 48, putative	At3g53230	Bra006978	7.6
		Bra003123	2.5
FAD-binding domain-containing protein	At1g26420	Bra024717	6.0
UDP-glucosyl transferase family protein	At1g22400	Bra012324	4.1
		Bra012323	3.7
Embryo-abundant protein-related	At3g54150	Bra007051	22.0
		Bra014837	1.1
Expressed protein	At1g13340	Bra016733	11
		Bra019826	1.4
Mannitol dehydrogenase, putative (ELI3-2)	At4g37990	Bra010627	9.9
WRKY family transcription factor	At5g13080	Bra008858	15
		Bra006178	9.5
FAD-binding domain-containing protein	At1g26380	ND	
Cytochrome P450 71B15, putative	At3g26830	ND	
Cytochrome P450, putative	At4g37370	Bra017819	8.8
bHLH transcription factor	At1g10585	Bra031721	6.3
Expressed protein	At4g39670	Bra004321	43.5
		Bra010670	1.4
Zinc finger (AN1-like) family protein	At3g28210	Bra025324	38
Glutathione S-transferase, putative	At2g29460	Bra039982	1.8
		Bra018385	1.4
Expressed protein	265674_at	ND	
2-oxoacid-dependent oxidase,	At3g49620	Bra017969	0.18

Transcripts representing ROS-induced genes in at leaset six out of eight stress experiments in *Arabidopsis* reported by Gadjev et al. [17] are listed. Fold change values of the space-grown Mizuna genes associated with the listed *Arabidopsis* genes are shown. ND, not detected.

ROS-scavenging genes are induced to defend the cells from oxidative stress. The expression level of transcripts associated with *Arabidopsis* ROS-scavenging genes [18] was confirmed in the space-grown Mizuna (Additional file 3: Table S3). In fact, 20 transcripts were up-regulated more than two-fold, including the major ROS-scavenging enzymes: superoxide dismutase, catalase, and glutathione peroxidase. A dramatic induction occurred in thioredoxin (Bra014037) and glutaredoxin (Bra030102, Bra032911) genes, of which the levels were increased more than 40-fold. Thioredoxin and glutaredoxin belong to the thioredoxin superfamily known to be crucial for maintaining a reduced intracellular redox state and oxidative defense [19]. Alternative oxidase (AOX) (Bra010153) was another highly expressed gene, with 9.2-fold up-regulation. AOX activity increases in plants under oxidative stress such as drought and salinity, and AOX functions as an antioxidant enzyme, acting to alleviate ROS accumulation [20-22]. These results suggest that the Mizuna modulates ROS-scavenging genes to eliminate oxidative stress induced by the spaceflight environment.

### Expression profiles of ROS-responsive transcription factor genes

**Figure 2:**
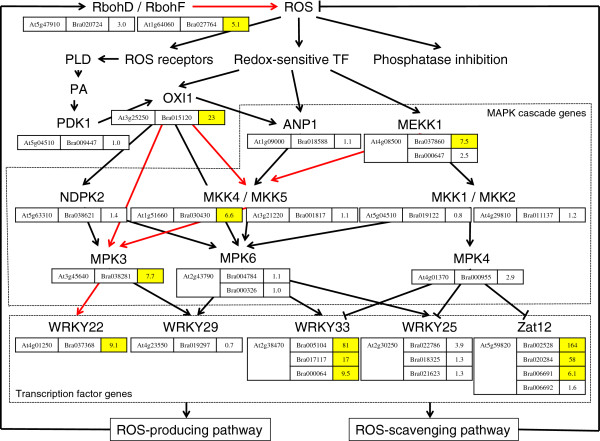
**Expression levels of genes in the space-grown Mizuna associated with MAPK cascade and transcription factor genes in ROS gene network.** Pathway of MAPK cascade and transcription factor genes in *Arabidopsis* known to activate under abiotic and biotic stress are shown. Genes and fold change values in the space-grown Mizuna associated with MAPK cascade and transcription factor genes in *Arabidopsis* are indicated in the boxes. The more than five-fold up-regulated genes are highlighted by yellow and predicted active MAPK cascades in the space-grown Mizuna are shown by red arrows.

Plants sense the ROS level and change the expression of genes comprising the ROS signal network that regulate growth, development, and stress defense. The ROS signals are induced via mitogen-activated protein kinase (MAPK) cascades, which play a crucial role in abiotic and biotic stress responses in plants (Figure 2). In *Arabidopsis*, MEKK1, OXI1, and ANP1 have been shown to function upstream to MAPK cascades. The MEKK1-MKK1/MKK2-MPK4 cascade was shown to a key regulator of ROS stress signaling [23] and an MEKK1-MKK2-MPK4/MPK6 cascade was identified in cold and salt stress [24,25]. An MEKK1-MKK4/MKK5-MPK3/MPK6 cascade was shown to function in protoplasts treated with bacterial flagellin peptide [26] and MKK4 regulated MPK3 under salt stress [27]. An ANP1-MKK4/MKK5-MPK3/MPK6 cascade was shown in H_2_O_2_ treated *Arabidopsis* plants [28]. An OXI1-MPK3/MPK6 cascade was activated by ROS [29] and an OXI1-NDPK2-MPK3/MPK6 was suggested to enhance tolerance against freezing and salinity [30]. Aside from the induction of MEKK1, ANP1, and OXI1 by H_2_O_2_, OXI1 is activated by PDK1 via the PLC/D-PA pathway. The expression levels of the genes in the space-grown Mizuna associated with *Arabidopsis* MAPK cascades are presented in Figure 2. MEKK1 (Bra037860), OXI1 (Bra015120), MKK4 (Bra030430), and MPK3 (Bra038281) were up-regulated more than five-fold, indicating that MEKK1-MKK4-MPK3, OXI1-MKK4-MPK3, and OXI1-MPK3 cascades were strongly activated via ROS under spaceflight environment. The expression of PDK1 gene (Bra009447) by 1.0-fold shows that PLC/D-PA-PDK1 pathway is not related to the up-regulation of OXI1 gene.

Transcription factor genes WRKY and Zat respond to oxidative stress to control the expression of ROS network genes, of which 11 WRKY and 3 Zat genes respond with more than five-fold expression by oxidative stress in *Arabidopsis*[17]. In the space-grown Mizuna, 7 WRKY and 2 Zat genes were shown to have more than five-fold expression (Table 2). Reportedly MEKK1-MEE4/MKK5-MPK3/MPK6 cascade activates WRKY22 and WRKY29 in the flagellin-treated *Arabidopsis*[26]. The expression levels of WRKY22 (Bra37368) and WRKY29 (Bra019297) in space-grown Mizuna were 9.1 and 0.7-fold, respectively, suggesting the activation of MEKK1-MKK4-MPK3-WRKY22 cascade. It was indicated to the negative control of WRKY25, WRKY33, and Zat12 by MEKK1-MKK1/MKK2-MPK4 cascade [23], however, WRKY25 (Bra022786), WRKY33 (Bra005104, Bra017117, Bra000064), and Zat12 (Bra002528, Bra020284, Bra006691) genes were up-regulated in the space-grown Mizuna, suggesting that the MEKK1-MKK1-MPK4 cascade in the space-grown Mizuna regulates other transcription factors or ROS network genes. Zat12 is the inducer of WRKY25 and Zat12 overexpression plants suppressed Zat7, which up-regulate WRKY70 [31,32]. Up-regulation of Zat12 and WRKY25 genes and lack of expression or down-regulation of Zat7 and WRKY70 genes in the space-grown Mizuna suggest that Zat12 is a regulator in the ROS signal transduction pathway involved in the spaceflight environment as well as abiotic and biotic stress.

**Table 2:** Expression of ROS-responsive transcription factor genes in the space- grown Mizuna

**Gene name**	**AGI code**	***B. rapa *** **gene**	**Fold change**
WRKY6	At1g62300	Bra027057	5.8
		Bra034482	1.1
WRKY18	At4g31800	Bra019123	43
		Bra023983	36
		Bra010220	26
		Bra011299	23
		Bra033532	1.9
		Bra032312	1.3
		Bra002913	1.1
WRKY25	At2g30250	Bra022786	3.9
		Bra018325	1.3
		Bra021623	1.2
WRKY30	At5g24110	Bra009734	4.1
		Bra026467	2.2
WRKY33	At2g38470	Bra005104	81
		Bra017117	17
		Bra000064	9.5
WRKY40	At1g80840	Bra008435	655
		Bra003588	161
		Bra035148	158
WRKY 43	At2g46130	ND	
WRKY48	At5g49520	Bra010032	64
		Bra036138	1.8
		Bra020628	0.8
WRKY53	At4g23810	Bra013732	26
		Bra019265	16
WRKY 70	At3g56400	Bra014692	0.78
		Bra003239	0.72
		Bra007243	0.36
WRKY75	At5g13080	Bra008858	15
		Bra006178	9.5
Zat7	At3g46070	ND	
Zat10	At1g27730	Bra010922	153
		Bra032845	45
Zat12	At5g59820	Bra002528	164
		Bra020284	58
		Bra006691	6.1
		Bra006692	1.6

Transcripts representing oxidative stress-induced transcription factor genes in *Arabidopsis* reported by Gadiev et al. [17] are listed. Fold change values of the space-grown Mizuna genes associated with the listed *Arabidopsis* genes are shown.

ND, not detected.

**Figure 3:**
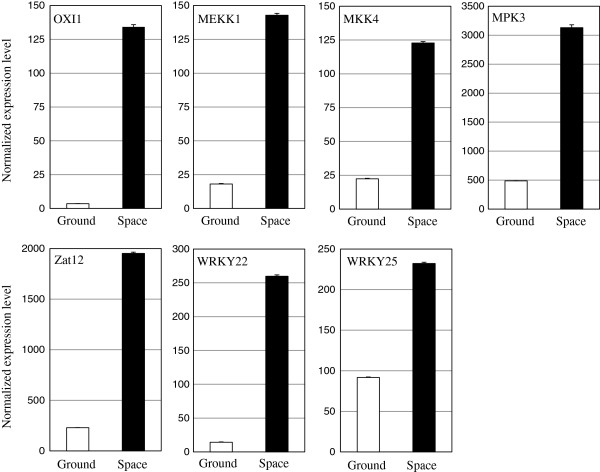
**Expression of the predicted active MAPK cascade and transcription factor genes in the space-grown Mizuna by qRT-PCR.** Expression levels of the predicted active genes, OXI1, MEKK1, MKK4, MPK3, Zat12, WRKY22 and WRKY25 in the space-grown and the ground-grown Mizuna were analyzed by qRT-PCR. Expression levels of genes in the space-grown and the ground-grown Mizuna were normalized with that of the α-tubulin gene as an internal control. The error bar represents standard error of the mean for three RNA isolation experiments.

To confirm the predicted active MAPK cascade and transcription factor genes in the space-grown Mizuna by mRNA-Seq, gene expression was analyzed by quantitative RT-PCR using gene-specific primers (Additional file 4: Figure S1, Additional file 5: Table S4). The expression levels of OXI1, MEKK1, MKK4, MPK3, Zat12, WRKY22 and WRKY25 were increased 38, 8, 5, 6, 8, 18 and 3-fold, respectively, in the space-grown Mizuna (Figure 3). This result correlates with that of mRNA-Seq, supporting the predicted active MAPK cascade and transcription factor genes under the spaceflight environment.

### Expression profiles of ROS-producing genes

Reactive oxygen species are used as a signal molecular in plants to amplify the ROS signal network to activate defense mechanisms against abiotic and biotic stress [18]. NADPH oxidases play a key role in producing ROS. RbohD and RbohF genes of NADPH oxidases in *Arabidopsis* were induced by oxidative stress, leading to maintain the stress defense system [18,33]. There were 7 NADPH oxidase genes identified in space-grown Mizuna and RbohD (Bra020724) and RbohF (Bra027764) genes were up-regulated by 3.0-fold and 5.1-fold, respectively (Table 3). This result suggests that space-grown Mizuna produces ROS to maintain the activation of ROS signal network to control the defense system with ROS-scavenging system.

**Table 3:** Expression of NADPH oxidase genes in the space-grown Mizuna

**Gene name**	**AGI code**	***B. rapa *** **gene**	**Fold change**
NADPH oxidase (RbohA)	At5g07390	Bra009266	1.1
NADPH oxidase (RbohB)	At1g09090	Bra031658	0.8
NADPH oxidase (RbohC)	At5g51060	Bra011911	ND
NADPH oxidase (RbohD)	At5g47910	Bra020724	3.0
NADPH oxidase (RbohE)	At1g19230	Bra025721	1.0
NADPH oxidase (RbohF)	At1g64060	Bra027764	5.1
NADPH oxidase (RbohG)	At4g25090	Bra019189	1.1
NADPH oxidase (RbohH)	At5g60010	Bra020270	ND
NADPH oxidase (RbohI)	At4g11230	Bra033151	1.1
NADPH oxidase (RbohJ)	At3g45810	Bra038274	ND

NADPH oxidase genes in *Arabidopsis* are listed and fold change values of the space-grown Mizuna genes associated with the listed *Arabidopsis* genes are shown. ND, not detected.

## Conclusions

Plants respond to environmental stress in multiple ways and have evolved mechanisms to increase their tolerance to the stress through interactive molecular and cellular changes. The components of these changes are responses of integrated ROS gene network triggered under stressful conditions. Our large-scale transcriptome analysis demonstrated that the spaceflight environment induced oxidative stress and activated a ROS gene network in the space-grown Mizuna, some of which were common genes up-regulated by abiotic and biotic stress and some of which were preferentially up-regulated genes by the spaceflight environment, even though Mizuna grew in the space as well as on the ground. Up-regulation of AOX, RbohD, and RbohF genes, activation of more than one MAPK cascades, and regulation of Zat7 and WRKY70 by Zat12 are common in plants under space, abiotic, biotic stress. Preferential high expression of thioredoxin and glutaredoxin genes, no repression of WRKY25, WRKY33, and Zat12 by MEKK1-MKK1/MKK2-MPK4 cascade, and no induction of OXI1 by PLC/D-PA-PDK1 pathway are different from those under abiotic and biotic stress. These results show that the ROS gene network for abiotic and biotic stress response and tolerance, which plants have developed to adapt the environment on the earth, could serve to acclimate plant to the abiotic stress conditions of the spaceflight environment.

## Methods

### Plant cultivation and spaceflight

Seeds of Mizuna, *Brassica rapa var. nipposinica*, were transported to ISS. Ten-15 seeds were set in each of two root modules of the Lada growth chambers aboard the Zvezda module of ISS [14,15], and allowed to germinate. Lada consists of a control module, a water reservoir and 2 growth chambers which include a root module, leaf chamber and light module (Additional file 6: Figure S2). The light banks house two cool white flourescent lamps providing approximately 125-250 μmol m^-2^ s^-1^ (depending on plant height) photosynthetically active radiation (PAR). In the overall Lada experiment, two root module growth substrate mixtures were used to determine which was optimal for plant growth. One mixture contained rinsed and dried arcillite (Turface Proleague, Profile LLC, Buffalo Grove IL, US) with 15 grams of 14-14-14 Osmocote fertilizer, the other, nutrient solution soaked and dried arcillite with 14 grams of a custom mixed Nutricote fertilizer. The Lada moisture level control point was set at 85% at the start of the experiment for both root modules. One of the root modules experienced an over-watering event in which the pump failed to stop when the moisture sensors reached 85% and almost 2 liters of water was added in 35 hours. Automatic watering was disabled and the pump was operated manually for the duration of the experiment. This was duplicated in the ground controls. Because of the difference in watering regimes, only the plant tissue from the Osmocote fertilized root modules with manually controlled watering, both flight and ground controls, were used for this study. The plants were grown in Lada under 24 hr lighting. The average environmental conditions measured from the Zvezda module of the ISS over the duration of the experiment and duplicated for the ground controls were 24.3 ± 0.8°C, 3.27 ± 0.46 mm Hg pp CO_2_, (= 4,303 ± 605 ppm CO_2_) and 40.7 ± 6.9% RH. The average recorded values for the ground control experiment were 24.5 ± 0.5°C, 4,254 ± 566 ppm CO_2,_ and 41.2 ± 2.6 RH. After 27 days of cultivation, the plants were harvested and stored at -80°C in the MELFI freezer onboard the Destiny module, and were transported to the ground at < -20°C in the GLACIER cold stowage system aboard the Space Shuttle. The total radiation dose during cultivation was 8.37 mGy.

### RNA preparation, mRNA-Seq analysis and identification of responsive transcripts

Total RNA from leaves samples was extracted using the RNeasy Plant Mini kit (Qiagen, Hilden, Germany) and cDNA library was constructed using the TruSeq^TM^ RNA sample preparation kit (Illumina, San Diego, CA, USA). Sequencing was performed on each library to generate 100-bp paired-end reads for transcriptome sequencing on Illumina Genome Analyzer IIx platform. Stretches of low quality bases at both sides of reads were trimmed using a customized C program (Q value < 15). Adapter sequences were removed using cutadapt (ver. 1.0; http://code.google.com/p/cutadapt/) with parameters ‘-e 0.1 -O 5 -m 20’ [34]. After pre-processing the Illumina reads, the transcript structures were reconstructed using a series of programs, namely, Bowtie 2 (ver. 2-2.0.0-beta7; http://bowtie-bio.sourceforge.net/bowtie2/index.shtml) for short-read mapping [35], TopHat (ver. 2.0.5; http://tophat.cbcb.umd.edu/) for defining exon-intron junctions [36], and Cufflinks (ver. 2.0.2; http://cufflinks.cbcb.umd.edu/) for gene structure predictions [37]. For TopHat, the Reference-*Brassica rapa* (Chiifu-401) genome (Phytozome; http://www.phytozome.net/) was used as the reference sequences with the following options: --no-discordant --no-mixed -r 46 --mate-std-dev 133 [38].

The expression level for each transcript was calculated as reads per kilobase of exon model per million mapped (RPKM) values based on the number of uniquely mapped reads that completely overlap with the exonic regions in each library [39]. The significantly differentially expressed transcripts between space- and ground-grown samples were detected by G-test (FDR < 0.001). Fold changes of expression levels between samples were calculated after adding one to each RPKM value to avoid division by zero.

### Gene ontology analysis

The GO terms assigned to the responsive transcripts were obtained from InterProScan 5 Release Candidate 2 (http://code.google.com/p/interproscan/) with the parameters ‘-f tsv -t p -dp –goterms’ for each transcript [40].

### GO enrichment analysis

GO enrichment was evaluated for responsive transcripts using Fischer’s exact test with a FDR threshold of 5% using R ver. 2.13.0 (http://www.r-project.org).

### Quantitative RT-PCR (qRT-PCR)

Poly(A)^+^ RNA was purified from total RNA with the Poly (A) Purist MAG (Ambion, Texas, USA) and 1st strand cDNA was synthesized from poly(A)^+^ RNA with the PrimeScript first strand cDNA Synthesis Kit (Takara Bio, Japan) according to the manufacture’s instruction. qRT-PCR was performed in a mixture of 10 μl containing 1st strand cDNA, SYBR Premix Ex Taq (Takara Bio, Japan), and 0.2 μmol each primer combination (Additional file 5: Table S4) using the LightCycler 2.0 (Roche Applied Science, Mannheim, Germany). The thermal cycle profile was 1cycle of 95˚C for 10 sec followed by 40 cycles of 95˚C for 5 sec, 60˚C for 20 sec. The cDNA quantities of each gene were calculated using LightCycler 4.0 software and the transcript expression levels were normalized with that of the α-tubulin gene as an internal control. Three technical replicate for each RNA were used for analysis. Specificity of primer combination was confirmed by melting curve of the PCR products and agarose gel electrophoresis (Additional file 4: Figure S1).

### Supporting data

The resulting mRNA-Seq data were deposited in the DDBJ Sequence Read Archive (http://trace.ddbj.nig.ac.jp/dra/index_e.html) under the accession No. DRA001106.

## Competing interests

The authors declare that they have no competing interests.

## Authors’ contribution

MS and YO contributed equally to the design and conduct of the experiments. MS, YO, OG, and TM arranged the mRNA-Seq experiment and discussed the results. MS, YO, and TY designed and performed data analysis. MS drafted the manuscript. MAL and VNS operated the cultivation in Lada aboard ISS. GEB, RW, and MH operated the transportation of plants from ISS to the ground and prepared the ground control plants. All authors have read and approved the final manuscript.

## Supplementary Material

Additional file 1: Table S1 Responsive transcripts in the space-grown Mizuna by G-test of the PRKM-derived read counts. Click here for file

Additional file 2: Table S2 Distribution of up-regulated and down-regulated genes in biological process category of Gene Ontology. Click here for file

Additional file 3: Table S3 Expression of ROS-scavenging genes in the space-grown Mizuna. Click here for file

Additional file 4: Figure S1 Agarose gel electrophoresis of the PCR products. The reaction mixture of PCR was loaded on agarose gel electrophoresis using 3% NuSieve 3:1 agarose with 1x TBE buffer and amplified fragments were detected by GelRed staining. M, 100 bp ladder marker. Click here for file

Additional file 5: Table S4 Primers for qRT-PCR. Click here for file

Additional file 6: Figure S2 Lada growth chamber aboard the Zvezda module of ISS. Lada consists of a control module, a water reservoir, and two growth chambers which include a root module, leaf chamber and light module. Click here for file
