# Supplementary figures and images for: Genome-wide expression analysis of reactive oxygen species gene network in Mizuna plants grown in long-term spaceflight
Source: BMC Plant Biol. 2014 Jan 6;14:4. doi: 10.1186/1471-2229-14-4 (PMC3927260; doi:10.1186/1471-2229-14-4)

## Slide 1
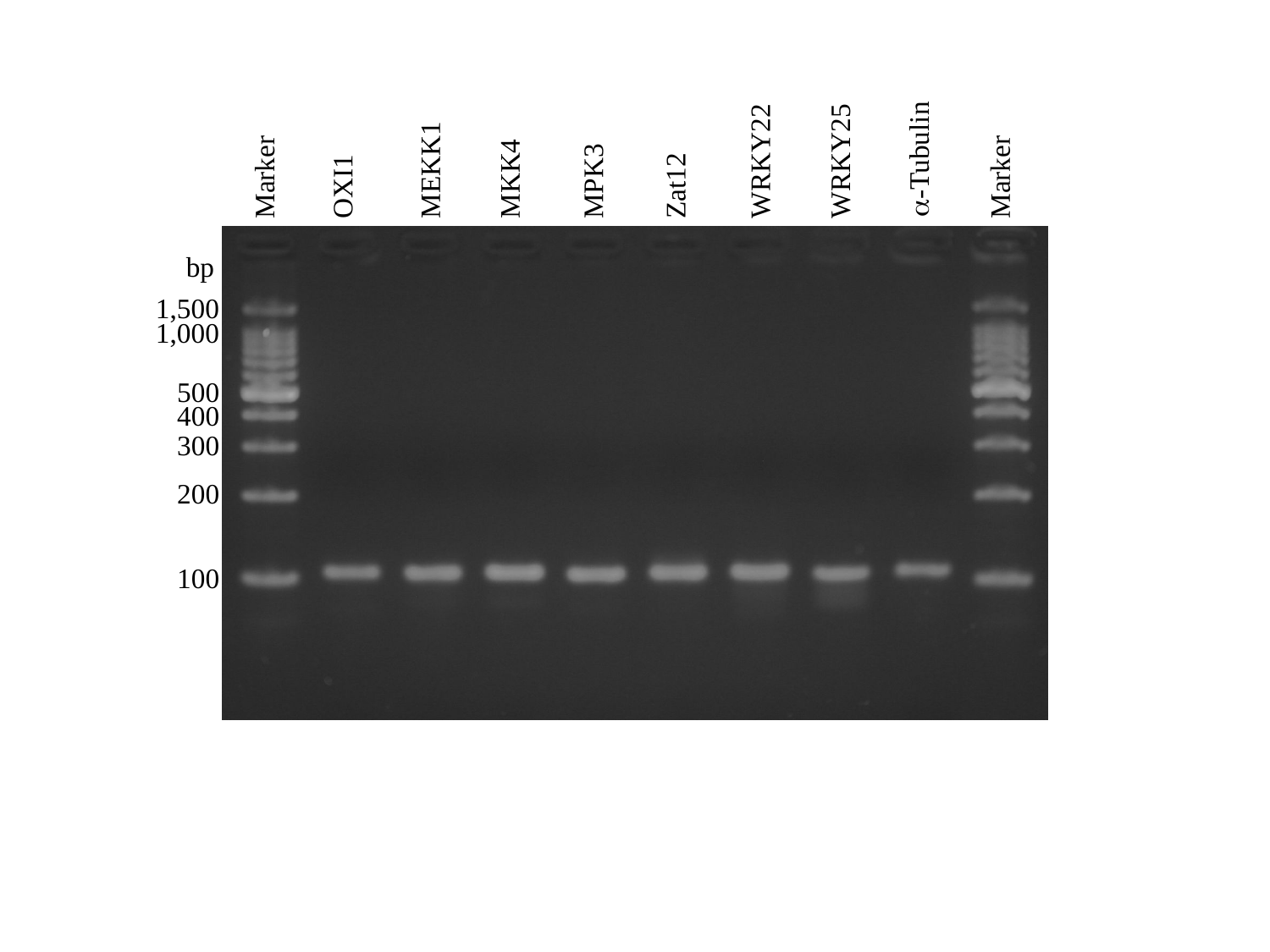

a-Tubulin
WRKY22
WRKY25
MEKK1
Marker
Marker
MKK4
MPK3
Zat12
OXI1
bp
1,500
1,000
500
400
300
200
100

Supplement: Additional file 4: Figure S1 — Agarose gel electrophoresis of the PCR products. The reaction mixture of PCR was loaded on agarose gel electrophoresis using 3% NuSieve 3:1 agarose with 1x TBE buffer and amplified fragments were detected by GelRed staining. M, 100 bp ladder marker. [file 1471-2229-14-4-S4.pptx]

## Slide 1
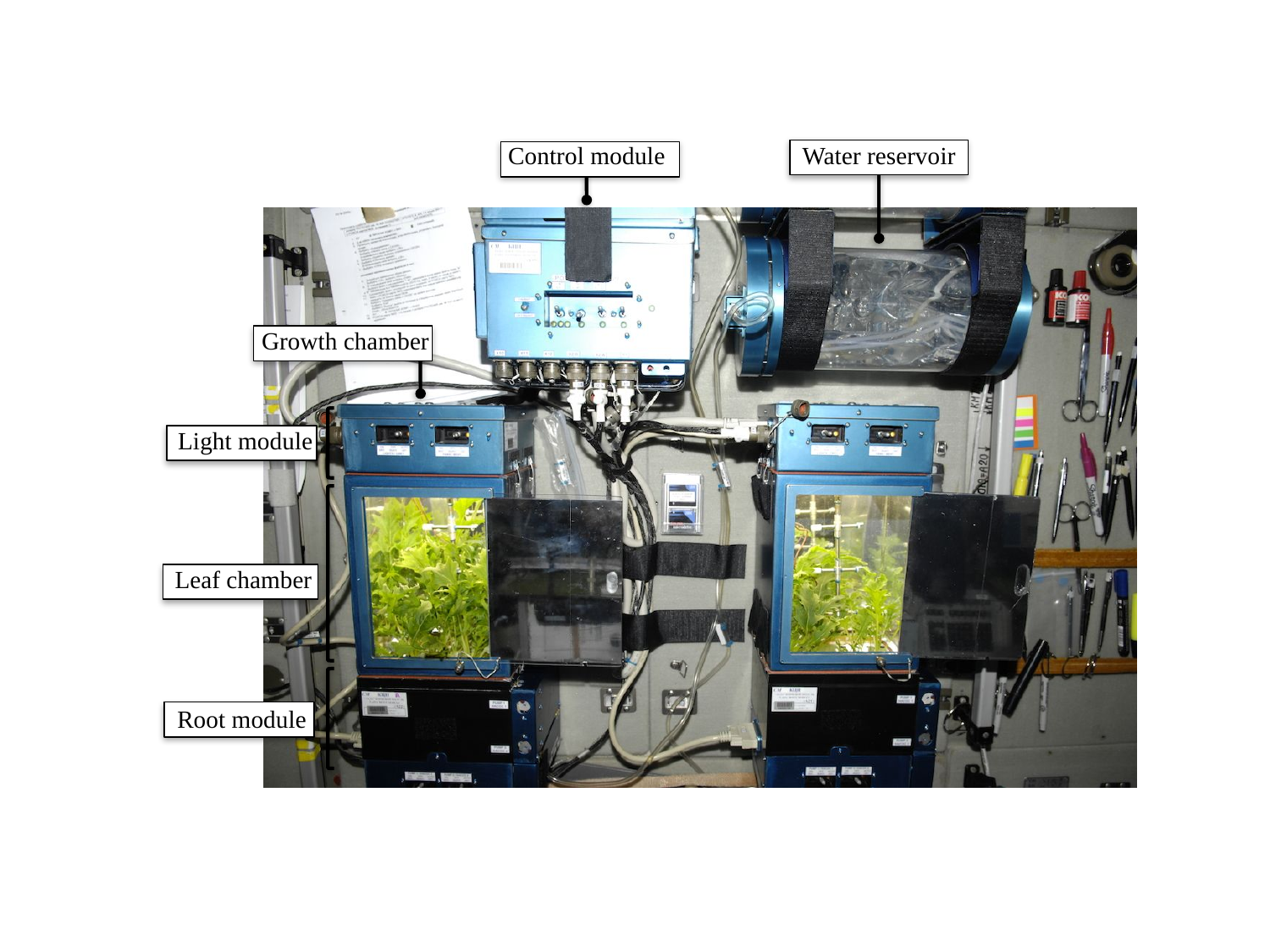

Control module
Water reservoir
Growth chamber
Light module
Leaf chamber
Root module

Supplement: Additional file 6: Figure S2 — Lada growth chamber aboard the Zvezda module of ISS. Lada consists of a control module, a water reservoir, and two growth chambers which include a root module, leaf chamber and light module. [file 1471-2229-14-4-S6.pptx]
